# Onlay mesh repair for treatment of small umbilical hernias ≤ 2 cm in adults: a single-centre investigation

**DOI:** 10.1007/s10029-021-02509-2

**Published:** 2021-09-30

**Authors:** M. Melkemichel, L. Stjärne, S. Bringman, B. Widhe

**Affiliations:** 1 Department of Surgery, Södertälje Hospital, 152 86 Södertälje, Sweden; 2 Department of Clinical Sciences, Danderyds Hospital, Karolinska Institutet, Stockholm, Sweden; 3 Department of Clinical Science and Education, Södersjukhuset, Karolinska Institutet, Stockholm, Sweden

**Keywords:** Small umbilical hernia, Mesh repair, Surgical site complication, Recurrence

## Abstract

**Purpose:**

Previous studies on the repair of small umbilical hernias have suggested a lower recurrence rate with mesh compared to suture repair. An important question is in what anatomical position the mesh should be placed. The purpose of this study was to investigate the outcome of using a standardized 4 × 4 cm onlay-mesh for umbilical hernias ≤ 2 cm.

**Methods:**

A retrospective study was conducted at a single centre in Sweden on all umbilical hernia repairs during 2015–2019. The follow-up time was at least four months. Patients were identified using the hospital medical database. Repairs performed with suture or a sublay, ventral patch and laparoscopic mesh positioning were excluded. The patient’s demographics, comorbidities, intra—and post-operative details were considered. The primary outcome was surgical site complications within 30 days. The secondary outcome was a recurrence.

**Results:**

80 patients were repaired with a small onlay-mesh for an umbilical hernia ≤ 2 cm. The median (range) follow-up time was 29.0 (4.3–50.1) months. The median age was 46 (26–76) years old. The median body mass index was 28 (19–38) kg/m^2^. The male to female ratio was 2:1. 4 patients were identified with a surgical site post-operative complication; three with seromas and one with a superficial wound infection. 3 of these were given antibiotics. 2 patients were treated with wound openings bedside. There were no registered cases of recurrence.

**Conclusions:**

Repairing small umbilical hernias with a small onlay-mesh was safe with a low surgical site complication rate. Randomized trials are needed to assess whether mesh can reduce recurrences in umbilical hernia repairs ≤ 2 cm.

## Introduction

The repair of small umbilical hernias in adults is common in Sweden’s general surgical practice [1]. Yet, a gold standard treatment is still not implemented. Traditionally, the surgical technique of choice has been either a Mayo repair or, more common, different variants of suture repair [2]. The recurrence rates with only a suture repair have not been negligible and have been described up to 20% [3]. Mesh repair has been usually reserved for larger umbilical hernia defects, but earlier reports show lower recurrence rates than suture repair also in smaller umbilical hernias [4–13]. Recently, the first treatment guidelines for small umbilical hernias recommended a pre-peritoneal flat mesh for the repair [14]. The recommendation could be considered based on Kaufman et al.’s recent large randomized clinical trial [15], whereas a pre-peritoneal mesh repair was compared to a simple suture repair. However, a sublay mesh placement could be difficult to implement easily without enlarging the defect in the case of small umbilical hernias. Also, the role of mesh in very small umbilical hernias < 1 cm remains uncertain. Consequently, an onlay-mesh placed above the sutured defect can be considered to achieve the same strength to retrain a hernia from recurring as with a sublay mesh placement, but at the same time be safer and more easily to perform in small defects. Despite the earlier reports of advantages with mesh reinforcement, there are still concerns about whether surgical site complications can become more frequent following a mesh repair. Therefore, the decision to use mesh needs to balance the risk of surgical site complications against the previous demonstrated reports of lower recurrence rates.

This study aimed to investigate a cohort of patients that had undergone a repair for a small umbilical hernia ≤ 2 cm with a standardized technique, using a small onlay-mesh. The hypothesis was that the rate of surgical site complications following the repair of using a small onlay-mesh in small umbilical hernia defects was low.

## Methods

### Study population

**Fig. 1:**
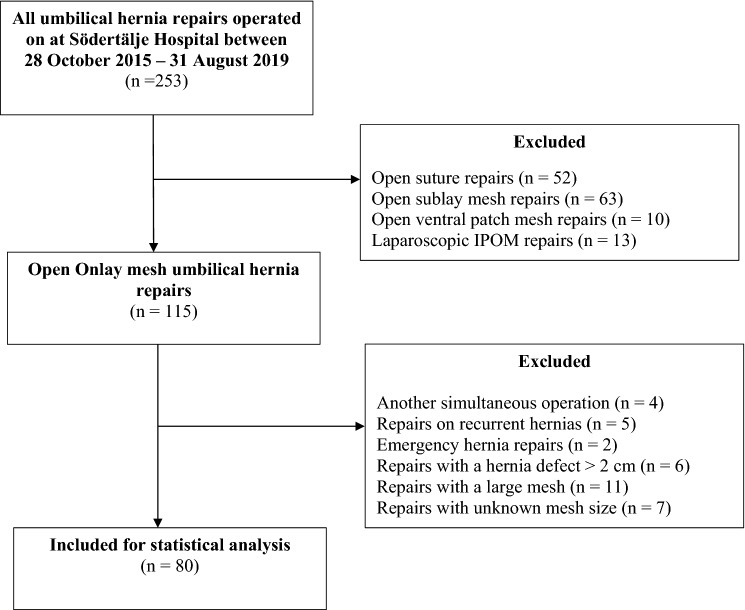
Flowchart of the included study population for statistical analysis; 80 open elective primary onlay umbilical hernia repairs ≤ 2 cm. *IPOM* intra-peritoneal onlay-mesh

The current study was carried out retrospectively using the hospital’s medical database after approval was obtained on 31 January 2020 by the Regional Ethics Review Board in Stockholm, Sweden (DNR: 2019-05608). All the patients that had undergone a repair for an umbilical hernia at the Department of Södertälje Hospital in the Region of Stockholm between 28 October 2015 and 31 August 2019 were identified using the International Classification of Diseases (ICD) code. Umbilical hernia repairs performed with a suture repair or a sublay, ventral patch and a laparoscopic mesh positioning were excluded. The numbers are presented in Fig. 1. Likewise, emergency repairs, repairs with another simultaneous operation, hernia defects > 2 cm and hernias repaired with a large onlay-mesh or an unknown mesh size were also excluded. Since this study aimed to investigate the outcome of using a small onlay-mesh repair on small hernias, the hernia repairs included in the statistical analysis had all a defect size ≤ 2 cm. The hernia defect size was measured intraoperatively by the surgeon and registered in the patient’s medical record. Incisional hernias that were wrongfully classified as primary umbilical hernias were identified and excluded. Clinically important variables that could affect the assessed outcomes, such as; demographics, comorbidities, intraoperative—and post-operative details, were collected from the patient’s medical chart database. These variables are presented in Table 1. The comorbidities that were noted was diabetes and chronic obstructive pulmonary disease (COPD). Immunosuppression, oral anticoagulants and smoking were selected as risk factors for the assessed outcome of the local surgical site complications. Moreover, the level of surgical competence, i.e., trainee (resident in general surgery) or consultant, was noted. Residents in Sweden can usually perform small umbilical hernia repairs independently after previous approval from their supervising consultants.

**Table 1:** Baseline hernia characteristics

	Onlay mesh repair of umbilical hernias (*n* = 80)
Age (years)	46 (26–76)
Follow-up time (months)^a^	29.0 (4.3–50.1)
Sex	
Male	54 (67.5)
Female	26 (32.5)
BMI (kg/m^2^)	28 (19–38)
ASA class	
I	21 (26.3)
II	50 (62.5)
III	9 (11.3)
IV	0
Preoperative imaging (yes)	29 (36.3)
Operation time (min)	41 (21–97)
Consultant (yes)	49 (61.3)
Risk factors	
Smoking	18 (22.5)
Immunosuppression	3 (3.8)
Anticoagulation	2 (2.5)
Diabetes	5 (6.3)
COPD	2 (2.5)

Data are in *n* (%) or in median (range)

*BMI* body mass index, *ASA class* American Society of Anesthesiologists, *COPD* chronic obstructive pulmonary disease

^a^The follow-up was the time since surgery whereas patients were followed in the outpatient clinical documentation for the treatment outcomes

### Study objectives

The primary outcome assessed was surgical site complication within 30 days after surgery. Patients were investigated for the treatment outcome of surgical site complications retrospectively via the hospital’s outpatient clinic documentation. All patients were carefully informed to directly contact the outpatient clinic at the department of Surgery at Södertälje Hospital if any postoperative complication was suspected. Patients were not contacted specially after surgery for a compulsory clinical examination A hematoma, a seroma and a wound infection were of most interest to investigate. A seroma was defined as an accumulation of clear fluid in the surgical field. A hematoma was defined as an accumulation of blood in the wound area. An infection was defined as a surgical site infection (SSI). The post-operative complications were graded according to the Clavien–Dindo classification [16]. The advent of the secondary outcome of recurrence was also investigated retrospectively from the outpatient clinic documentation. The definition of a recurrence was a bulging of a hernia (presented in the outpatient clinical setting) in the earlier performed umbilical repair site. All the included hernia repairs in this cohort were followed after surgery in the outpatient clinic documentation for the treatment of outcomes of site surgical complications and recurrence until 31 December 2019.

### The onlay-mesh repair

In 2015, in the Department of Surgery at Södertälje Hospital, a standard protocol was set up to treat ventral hernias. It was recommended that all the surgeons should use a small onlay-mesh repair for small umbilical and epigastric hernia defects ≤ 2 cm. If the defect size was considered to be larger than 2 cm, another mesh repair technique was recommended. All the surgeons were given a demonstration of the surgical technique by the involved authors concerning the onlay-mesh repair. It was described to be performed as an open incision in the umbilical area and a dissection of the hernia sac. The largest hernia defect diameter was recommended to be measured with a ruler intraoperatively and noticed in the patient’s medical record. Then the surgeon was recommended to perform a suture repair, in the transversal direction, with a continuous non-absorbable monofilament suture 2/0 of the aponeurosis defect. The subcutaneous tissue was then dissected from the aponeurosis so that the surgeon could apply a 4 × 4 cm (± 1 cm) Ultrapro^®^ or an Ultrapro Advanced™ (Ethicon Inc, part of the Johnson&Johnson family of companies, Germany) mesh to the site of the defect that had been closed. The mesh was recommended to be fixated with a single non-absorbable monofilament suture 2/0; one in the centre of the mesh and one in each corner in a transversal direction to prevent the risk of nerve-entrapment. In total, five single sutures were attached to the mesh. Both Ultrapro^®^ and Ultrapro Advanced™ are lightweight composite polypropylene meshes with an absorbable monofilament poliglecarpone-25 component. An absorbable monofilament suture was recommended to affix the umbilical skin to the aponeurosis. If the hernia involved the stalk, it was usually detached. The residual of it was then re-attached with an absorbable monofilament suture to the aponeurosis with the onlay-mesh. All the repairs were performed under general anesthesia. Antibiotics were usually not given preoperatively according to standard protocols. An illustrative picture of the onlay-mesh repair for small umbilical hernias can be found in the protocol of an ongoing trial (SUMMER Trial) for the treatment of small umbilical hernias in adults [17].

All the hernia repairs that distinguished from this onlay-mesh repair were excluded from the included analysis to ensure that all the included repairs were performed with somewhat the same standardized technique.

### Statistical analysis

A descriptive statistical analysis was carried out. Baseline characteristics of the included study population are described in Table 1. Data are presented in numbers and percentages. Continuous variables are presented in median with range. The frequency of local complications is presented in Table 2. In addition, a descriptive subgroup analysis of the onlay hernia repairs with local post-operative complications compared to the onlay hernia repairs without local complications is presented in Table 3. All analyses were performed using R Core Team 2017 with descriptive packages (Version 3.4.1. R: A language and environment for statistical computing. R Foundation for Statistical Computing, Vienna, Austria. URL https://www.R-project.org/).

**Table 2:** Outcomes of postoperative complications

	Onlay mesh repair of umbilical hernias (*n* = 80)
Local complications^a^	4 (5.0)
Seromas	3
Haematomas	0
Wound infections	1
Mesh infections	0
Wound openings bedside	2
Postoperative oral antibiotics	3
Systematic complications	
Urinary tract	0
Cardiovascular	0
Pulmonary	0
Recurrences^b^	
Yes	0
Reoperation within 30 days	0

Data are in *n* (%)

^a^Local complications were classified according to the Clavien–Dindo system; grade 1: 2; grade 2: 3; grade 3a: 0; grade 3b: 0; grade 4: 0; grade 5: 0

^b^Until the follow-up time

**Table 3:** Onlay hernia repairs with postoperative local complications compared to onlay hernia repairs without local complications

	Local complications (*n* = 4)	Without local complications (*n* = 76)
Age (years)	38 (34–56)	46 (26–76)
Follow-up time (months)	31.7 (21.8–50.1)	29.0 (4.3–50.1)
Sex		
Male	2 (50)	52 (68.4)
Female	2 (50)	24 (31.6)
BMI (kg/m^2^)	25 (19–34)	28 (19–38)
ASA class		
I	0	21 (27.6)
II	4 (100)	46 (60.5)
III	0	9 (11.8)
IV	0	0
Preoperative imaging (yes)	3	26 (34.2)
Operation time (min)	41 (33–64)	41 (21–97)
Consultant (yes)	2 (50)	47 (61.8)
Risk factors		
Smoking	2 (50)	16 (21.1)
Immunosuppression	0	3 (3.9)
Anticoagulation	0	2 (2.6)
Diabetes	0	5 (6.6)
COPD	0	2 (2.6)

Data are in *n* (%) or in median (range)

*BMI* body mass index, *COPD* chronic obstructive pulmonary disease

## Results

A total of 253 umbilical hernia repairs were registered in the hospital’s database with the ICD-codes of interest during this 4-year study period. After exclusion criteria, 80 elective primary small umbilical hernias ≤ 2 cm, repaired with a 4 × 4 cm (± 1 cm) onlay-mesh were remained for the statistical analysis.

### Baseline characteristics

All the baseline characteristics are given in Table 1. The mean age of the analyzed study population was 46 years old, with a clear overrepresentation of men. The median follow-up time was 29.0 months with a range of 4.3 months to 50.1 months. The median body mass index (BMI) was 28 kg/m^2^. All the patients were mostly healthy, classified as ASA class I or II. Preoperative imaging, either with computer tomography (CT scan) or ultrasound, was carried out in about a third of the patients. Among the registered risk factors, smoking was the most frequently observed in over 20% of the patients. The median operation time for the onlay-mesh repair was approximately 40 min. Half of the repairs were performed by consultants and if not, repaired by residents in general surgery.

### Assessed outcomes

4 out of 80 (5%) patients were identified with having a local surgical site complication (Table 2). A seroma was defined in three of these four repairs, and one case was defined as having a superficial wound infection. 3 of these four patients were prescribed oral antibiotics after surgery in the outpatient clinic. 2 of these three patients were also treated with a wound opening bedside and healed well. There were no cases of systemic complications. No cases of reoperation within 30 days were noted. There were no registered cases of recurrences until the follow-up period.

### Subgroup analysis

The onlay hernia repairs with a post-operative local complication were described compared to the onlay hernia repairs without a local complication for the subgroup analysis (Table 3). The group with the local complications were younger but with a lower median BMI. Operation time and the ASA class did not differ between the two groups. 50% (2 out of 4) of the repairs with local complications were smokers, compared to 21% (16 out of 76) of those without a local complication.

## Discussion

The results in this study supported our hypothesis and showed a low surgical site complication rate after repairing small umbilical hernias with a small onlay-mesh technique. The long-term follow-up did not either reveal any severe complications or reoperations for these patients. Additionally, not a single case of recurrence in this group of 80 patients during a total follow-up of 4 years was noteworthy.

To our knowledge, surgeons in Sweden have remained reluctant to use mesh in small umbilical hernias. A possible explanation could be due to different questions relating to the anatomical placement of the mesh in these small defects and concerns of higher risk of post-operative surgical site complications. With its limitations in consideration, this report can add important knowledge of the observed low surgical site complication rate of an onlay-mesh repair in small umbilical hernias.

When comparing our rate of surgical site complications to other studies, difficulties may arise since different definitions of complications are used. Also, different mesh positioning has been used in umbilical hernia repairs. This factor can certainly affect the following surgical site complication rate. A meta-analysis found an increased risk of seroma and surgical site infection (SSI) in the mesh group (7.3% SSI rate and 7.7% seroma rate) compared to the suture repair group (6.6% SSI rate and 3.8% seroma rate) [18]. In contrast, another meta-analysis indicated a clear benefit of using a mesh repair in reducing recurrence rates without any significant differences in surgical site complication rates between a mesh and a suture repair [19]. However, this analysis included only three randomized trials and only six of ten observational studies reported complication rates.

In contrast, the hernia size was found to be a clear confounder that was not adjusted for. Similar, Kaufman et al. reported a surgical site complication rate for the mesh group that did not significantly differ from the sutured repair group [15]. Still, the rate was not negligible in this large randomized trial and was described in 6.8% (10 out of 146) of the pre-peritoneal mesh repairs. Thereafter, a recent systematic review and meta-analysis of five randomized clinical trials, demonstrated again lower recurrence rates with mesh compared to a suture repair without any significant difference for surgical site complications of seroma, infection and hematomas between the groups [20]. However, the authors concluded that there were insufficient data to draw any robust conclusions regarding the outcome of surgical site complications.

Taken together, the presence of surgical site complications could seem to be slightly higher in the above-mentioned earlier reports than ours. The explanation could be that the analyzed studies are heterogeneous to hernia size and other factors as mesh positioning. The risk of developing a seroma is higher in larger hernia defects repaired with a retro-muscular technique, rather than in very small defects, repaired with a small onlay-mesh with a minor underlying subcutaneous dissection for the inserted mesh above the aponeurosis. Therefore, the onlay-mesh repair could be an operative technique of choice to reduce the high risk of recurrence in small umbilical hernias without increasing the risk for other surgical complications.

Furthermore, BMI can be one of the main factors that can affect the outcome of postoperative complications following a ventral hernia repair [21, 22]. The patients in this cohort had an average BMI of 28 kg/m^2^ and this could be one of the reasons for a low rate of surgical site complications. The explanation of the average BMI in the cohort could be that surgeons at the Department of Södertälje Hospital was not prone to perform surgery on patients with a high BMI for a small umbilical hernia. The symptoms of an elective small umbilical hernia are considered often to be benign and surgery is not necessary to perform immediately. If the patient presents a high BMI, usually the recommendation is to encourage patients to preoperative weight loss prior to open ventral hernia repair due to reduce the risk of postoperative complications.

Moreover, the risk of developing mesh-related infection seems to be very uncommon. After an open small umbilical hernia repair, either with or without a mesh used was described as low as 1–4% [11]. In our study, only 1 out of 80 patients was defined with a wound infection at the surgical site and was treated well with antibiotics at the outpatient clinic. However, two out of three patients with seromas were also treated with per oral antibiotics following surgery. In these cases, a probable explanation for prescribing antibiotics was considered due to fear of potential mesh infection.

This report does not address other post-operative complications, such as pain following the hernia repair. However, we did not observe any registered cases in the outpatient clinic documentation for post-operative pain during this study period. Also, a concomitant significant rectus diastasis with a small umbilical hernia was not investigated in this study. One explanation to this was that the patients that underwent an onlay-mesh repair for these small umbilical hernias in this cohort were considered to not likely have a concomitant significant rectus diastasis. The Department of surgery at Södertälje Hospital is one of the “hernia centers” in the Region of Stockholm and the surgeons at the department are well known with other treatment recommendations than a small onlay-mesh repair for patients with concomitant significant rectus diastasis.

There are some limitations with the study that can affect the interpretation of the results. Firstly, we are aware that the study population is limited and, as an effect of it, the rare events of the assessed outcomes. Therefore, it can be difficult to draw any conclusions regarding the potential cause or risk factors of the surgical site complications. Secondly, the report is only a descriptive analysis of the outcome of one method of choice. To assess whether a mesh repair in smaller umbilical hernias reduces the risk of recurrence without significantly increasing surgical site complications, a compared randomized control trial would be suitable. The authors have conducted an ongoing large randomized controlled trial with a sufficient amount of trial participants to investigate the outcome of the treatment of small umbilical hernias with either an onlay-mesh repair or a simple suture repair [17]. Thirdly, the study design of retrospectively collecting data and assessing the outcome of the patient’s medical record can certainly introduce bias in the cohort. Some of the adverse events of surgical site complications could have been missed regarding patients who decided to seek for a complication following their hernia repair in another hospital or their family doctor. Furthermore, missing data on the mesh size opt for exclusion of some of the hernia repairs. Finally, we observed that surgeons repaired some umbilical hernia defects > 2 cm with larger onlay-mesh. This situation led to a significant number of patient’s excluded from the analyzed study population. The selection was done to include a homogenous study population that represented only small onlay-mesh repair for small primary umbilical hernias.

In conclusion, this study can provide additional knowledge to surgeons considering using mesh in small umbilical hernia repairs. The study demonstrated a low surgical site complication rate following an onlay-mesh repair for small umbilical hernia defects ≤ 2 cm. An onlay-mesh repair can be a safe and easy alternative when choosing a repair method for the treatment of small umbilical hernias. However, it calls for further studies comparing the method with other repairs to assess whether an onlay-mesh can reduce recurrences in small umbilical hernia repairs without increasing the surgical site complication rate.

## Data Availability

All the collected data are uncoded and available in an excel file. It will also be available at Karolinska Institute’s electronic notebook database and can be provided on request.
